# The microbiota-gut-kidney axis mediates host osmoregulation in a small desert mammal

**DOI:** 10.1038/s41522-022-00280-5

**Published:** 2022-04-04

**Authors:** Zahra Nouri, Xue-Ying Zhang, Saeid Khakisahneh, Abraham Allan Degen, De-Hua Wang

**Affiliations:** 1 State Key Laboratory of Integrated Management of Pest Insects and Rodents, Institute of Zoology, Chinese Academy of Sciences, Beijing, 100101 China; 2 CAS Center for Excellence in Biotic Interactions, University of Chinese Academy of Sciences, Beijing, 100049 China; 3 Desert Animal Adaptations and Husbandry, Wyler Department of Dryland Agriculture, Institutes for Desert Research, Ben Gurion University of the Negev, Beer Sheva, 8410500 Israel; 4 School of Life Sciences, Shandong University, Qingdao, 266237 China

**Keywords:** Applied microbiology, Biofilms

## Abstract

Regulating sodium and water balances is crucial for survival of small, desert mammals. Studies demonstrate that the gut microbiota and their metabolites are involved in host energy homeostasis, but little is known on the interactions among salt loading, gut microbiota, and osmoregulation. The aim of this study was to fill this gap. Mongolian gerbils (*Meriones unguiculatus*) were offered drinking water (Con) and either water containing moderate (4%, MS) or high NaCl (8%, HS) ad libitum. Intake of HS reduced α diversity of the microbial community and, at the genus level, reduced the relative abundances of *Rikenella* and *Christensenella* but increased *Atopobium*. To confirm the function of gut microbiota in host osmoregulation, we transplanted caecal microbiota in HS gerbils. To cope with salt loading, the gerbils concentrated urine, resulting in negative energy balance and systemic inflammation. The HS gerbils increased hypothalamic arginine vasopressin and intestinal and renal aquaporin 2 to support water retention, and reduced intestinal and renal epithelial sodium channel α to promote sodium excretion. However, HS gerbils with caecal microbiota transplant (CMT) from Con donors maintained energy balance and osmoregulation, and had a much reduced systemic inflammation. Further, CMT from Con donors to HS recipients reshaped the gut microbiota, particularly by reducing *Parabacteroides distasonis* and *Prevotella copri*, and increasing *Lactobacillus reuteri* abundances, with a resulting increase in bacterial metabolites such as butyrate. These findings highlight a vital role of the microbiota-gut-kidney axis in mediating salt-related osmoregulation, allowing small mammals to adapt to high salt loads in a desert habitat.

## Introduction

High salt consumption in the modern world has caused numerous disorders, including hypertension, cardiovascular diseases, kidney damage, and cognitive impairment in human and laboratory animal models^1^. Desert mammals have evolved strategies to cope with water shortage and concentrated electrolytes in vegetation^2^. These mammals rely heavily on concentrating their urine and reabsorbing renal water to conserve body water in response to high salt loads^3^. Osmoregulation is mediated mainly by neural and hormonal pathways, for example by the regulation of arginine vasopressin (AVP), which is synthetized in the paraventricular and superoptic nuclei of the hypothalamus and released from the pituitary to induce translocation of aquaporin 2 (AQP2) water channels to the plasma membrane^4^. In addition, the epithelial sodium channel α (α-ENaC), present in membranes of epithelial cells and regulated by hormones such as aldosterone, modulates the amount of Na^+^ in the extracellular fluid and also blood pressure^5^.

The Mongolian gerbil (*Meriones unguiculatus*), a social rodent, inhabits desert grasslands and agricultural fields in northern China, Mongolia, and Russia^6^. This small mammal can feed on plants containing up to 10% sodium content^7^. This species has a wide thermoneutral zone and is able to survive air temperature of 37 ^o^C for >3 weeks without brain oxidative damage^8,9^. These gerbils also display efficient water conservation mechanisms, including a wide relative medullary thickness, increasing expression of renal aquaporin 2 (AQP2) for water reabsorption and highly concentrated urine^10^. In addition, they reduce serum aldosterone levels and renal epithelial sodium channel (α-ENaC) expression to promote sodium excretion in response to high salt intake^11^.

The gut microbiota are sensitive to inner and outer environments and play critical functions in the host’s energy homeostasis. For example, dynamic or seasonal fluctuations in air temperature and diet induced transient or persistent alterations in the gut microbiota in Brandt’s voles (*Lasiopodomys brandtii*)^12^, Mongolian gerbils^13^ and yaks (*Poephagus grunniens*)^14^. Studies in mice demonstrated that salt ingestion led to loss of *Lactobacillus* strains, which causes hypertension and autoimmunity^15^. Short chain fatty acids (SCFAs), as key bacterial metabolites, act on G-protein-coupled receptors (free fatty acid receptor 2, FFAR2), regulate the secretion of gastrointestinal hormones, such as cholecystokinin (CCK), ghrelin and leptin, which are involved in the regulation of food intake, metabolic rate and body weight^16–18^. In addition, butyrate activates the cAMP-PKA-CREB signaling pathway and stimulates Na^+^-H^+^, Cl^−^-SCFA and Cl^−^-HCO_3_^−^ exchanges across the apical membrane to absorb electrolytes by the colon^19–21^.

However, whether gut microbiota play a role in the regulation of host salt/water homeostasis is unknown. The aim of this study, at least in part, was to fill this gap. We hypothesized that the interaction between gut microbiota and host kidney mediated osmoregulation in desert rodents with high salt intake. To test this hypothesis, we investigated the impact of salt intake on the microbiota community and responses related to osmoregulation, and then determined the function of the gut microbiota in host sodium and water balances using Mongolian gerbils as a model.

## Results

### Salt intake altered faecal microbiota profile and bacterial metabolites

**Fig. 1:**
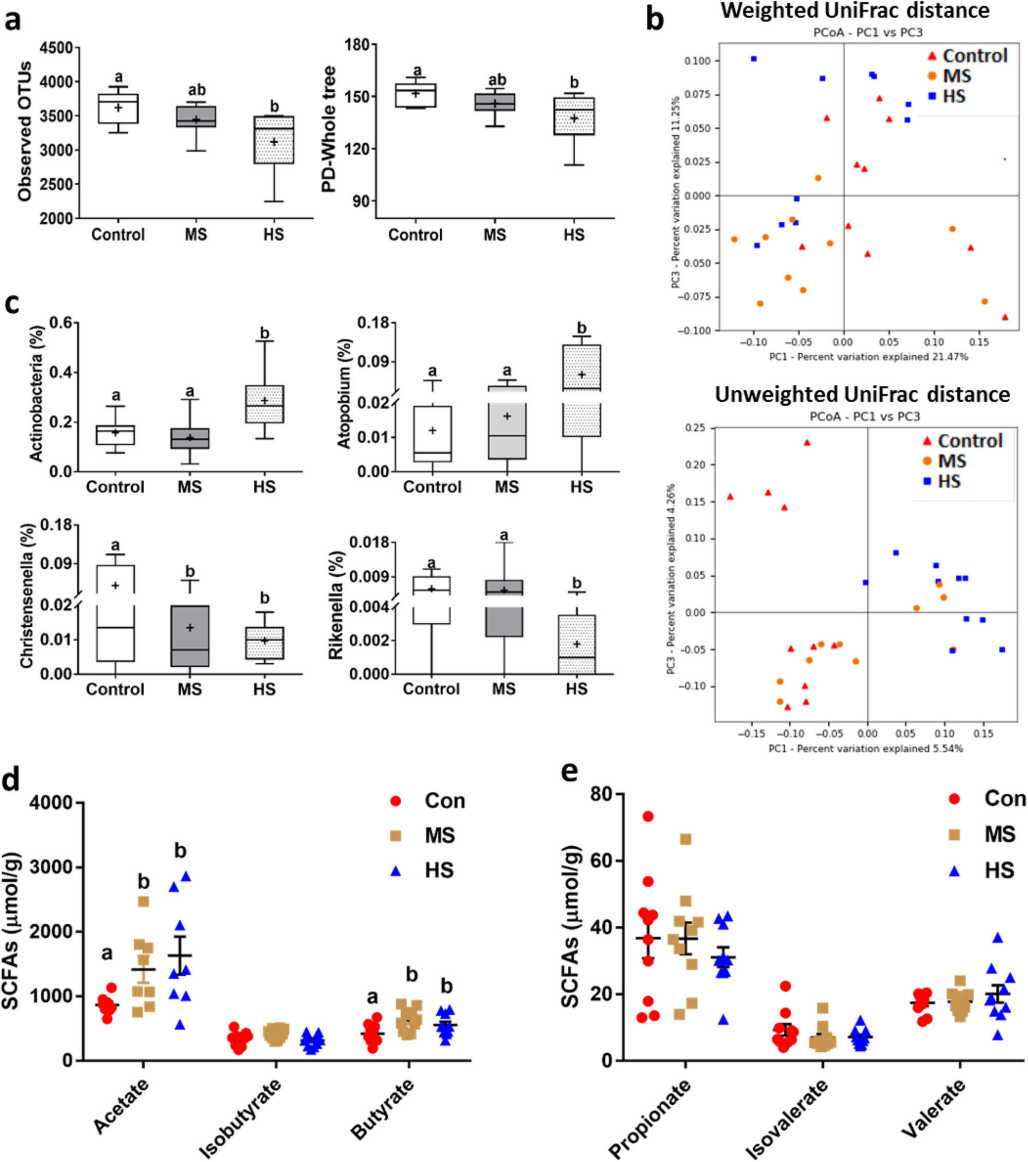
Salt intake altered faecal microbiota. Alpha diversity indicated by observed operation taxonomic units (OTUs, *P* = 0.005), and phylogenetic diversity (PD) whole tree (*P* = 0.013) (**a**); principal coordinate analyses (PCoA) plots based on weighted (*P* = 0.001) and unweighted (*P* = 0.001) UniFrac distances in faecal microbiota of different groups (**b**); relative abundances of different bacteria at the phylum and genus levels in different experimental groups (“+” indicates the mean value) (**c**); and concentrations of different short-chain fatty acids (SCFAs) (**d**, **e**) in control (Con), medium salt (MS), and high salt (HS) gerbils. Data are presented as means ± standard error of the mean (s.e.m), and the bars which do not share the same letter are significantly different from each other (*P* < 0.05).

The gerbils were offered drinking water ad libitum with either 0 (control, Con), 4% (medium salt, MS) or 8% NaCl (high salt, HS) for 4 weeks and faecal samples were collected at the end of salt acclimation to examine the effect of salt intake on the diversity and composition of gut microbiota and bacterial metabolites. The HS intake induced a reduction in α diversity of the microbiota community indicated by operation taxonomic units (OTUs) and phylogenetic diversity (PD) whole tree when compared to Con, but the MS and Con groups did not differ (Fig. 1a and Supplementary Table 1). The microbiota structure (β diversity), indicated by principal coordinate analyses (PCoA) based on weighted (ANOSIM, *R* = 0.436, *P* = 0.001) and unweighted (ANOSIM, *R* = 0.542, *P* = 0.001) UniFrac distances in the faecal microbiota communities, displayed clear separation among groups (Fig. 1b).

The dominant bacterial phyla (mean relative abundance > 5%) included Firmicutes (71.8%), Bacteroidetes (19.3%) and Proteobacteria (7.0%), and lesser phyla included Spirochaetes (1.1%), Cyanobacteria (0.1%), Actinobacteria (0.2%), and TM7 (0.3%) (Supplementary Fig. 1a). At the phylum level, the relative abundance of Actinobacteria was greater in the HS group than the Con and MS groups (*F*_2, 27_ = 8.869, *P* = 0.001; Fig. 1c). At the genus level, the relative abundance of *Atopobium* was greater in the HS group than the Con and MS groups (*F*_2, 27_ = 5.298, *P* = 0.011; Fig. 5c), but *Christensenella* (*F*_2, 27_ = 3.570, *P* = 0.042) and *Rikenella* (*F*_2, 27_ = 3.418, *P* = 0.047) were lower in HS than Con (Fig. 1c). To assess the effect of salt intake on microbial communities, we applied the LEfSe method with LDA score > 2 and identified 11 distinctive bacteria in Con, 4 in MS, and 12 in HS (Supplementary Fig. 1b, c). The concentrations of acetate (ANOVA, *F*_2, 27_ = 3.526, *P* = 0.048) and butyrate (ANOVA, *F*_2, 27_ = 4.260, *P* = 0.025) in faeces were greater in the MS and HS groups than in the Con group (Fig. 1d), and other SCFAs showed no group differences (Fig. 1d, e).

### Gut microbiota regulate host sodium and water balances

**Fig. 2:**
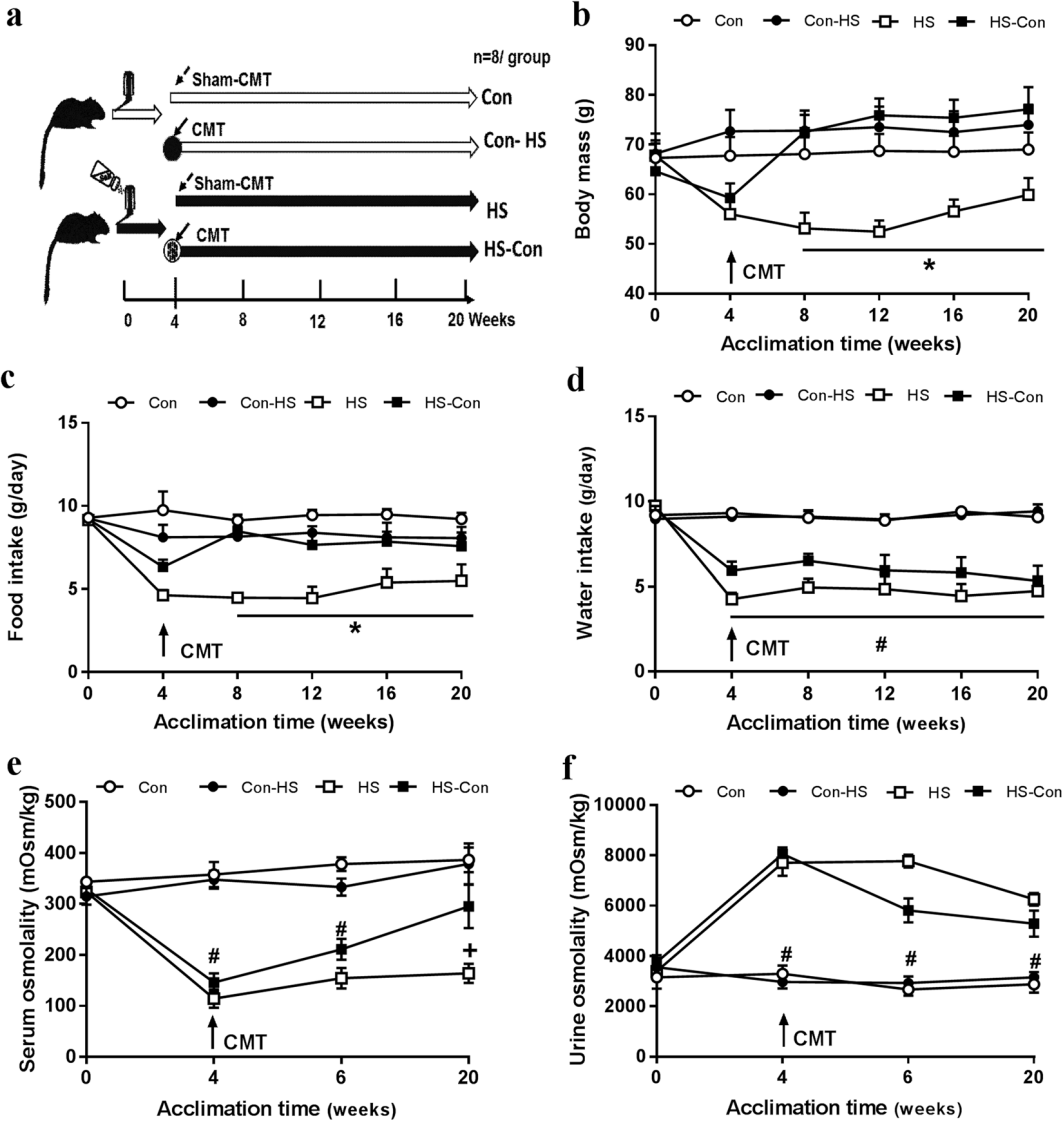
Microbiota transplant reversed salt-induced changes in energy balance and osmolality. Schematics of experimental design (**a**), body mass (**b**), food intake (**c**), water intake (**d**), serum osmolality (**e**), and urine osmolality (**f**) after caecal microbiota transplant (CMT). *P < 0.05, HS vs other groups (HS-Con, Con and Con-HS); ^+^*P* < 0.05, HS vs Con and Con-HS; ^#^*P* < 0.05, both HS and HS-Con vs Con and Con-HS. Data are means ± standard error of the mean (s.e.m). Con, gerbils drank tap water ad libitum and received sterile saline as sham-CMT via oral gavage; Con-HS, Con gerbils received microbiota from HS gerbils via oral gavage; HS, gerbils drank water with 8% NaCl ad libitum and received sterile saline as sham-CMT via oral gavage; HS-Con, gerbils drank HS water ad libitum and received microbiota from the Con gerbils via oral gavage.

To test the effects of salt intake and gut microbiota on host sodium and water balances, another set of gerbils received drinking water ad libitum with 8% NaCl (HS) or with no added NaCl (Con), and caecal microbiota was transplanted from control gerbils into half of HS gerbils (HS-Con) and from the HS group into half of the Con group (Con-HS) after 4-week HS intake. The gerbils received HS water or just water ad libitum for 20 weeks in total (Fig. 2a). The other half acted as sham caecal microbiota transplant (CMT) group (HS and Con) (Fig. 2a). Both body mass and food intake were lower in the HS than Con group (ANOVA, *P* < 0.001; Figs. 2b, c); however, these changes were recovered to the Con level in the HS-Con group. The CMT in the control gerbils from HS donors (Con-HS) did not alter either body mass or food intake. Salt intake reduced water intake (ANOVA, *F*_3, 28_ = 58.130, *P* < 0.001; Fig. 2d), but CMT from Con donors to HS recipients did not affect water intake.

Serum osmolality was lower in the HS than Con group in week 4 (ANOVA, *F*_3,28_ = 42.296, *P* < 0.001, Fig. 2e), consistent with the previous findings^11^, and the HS group maintained this low level till the end. However, CMT in HS gerbils from Con donors achieved serum osmolality homeostasis at the end of the 20-week acclimation (Fig. 2e). Urine osmolality was 1.3 fold higher in the HS (7703 ± 520.9 mOsm/kg) than Con (3297 ± 328.4 mOsm/kg) group in week 4 (ANOVA, *F*_3,28_ = 58.35, *P* < 0.001) and this high level was maintained till the end of the 20 weeks (7665 ± 1010.3 mOsm/kg, Fig. 2f); whereas CMT (HS-Con) buffered the HS-induced increase and had an intermediate urine osmolality in week 20 (5230 ± 521.9 mOsm/kg, *F*_3,28_ = 16.961, *P* < 0.001, Fig. 2f).

### Microbiota transplant alleviated HS-induced systemic inflammation

**Fig. 3:**
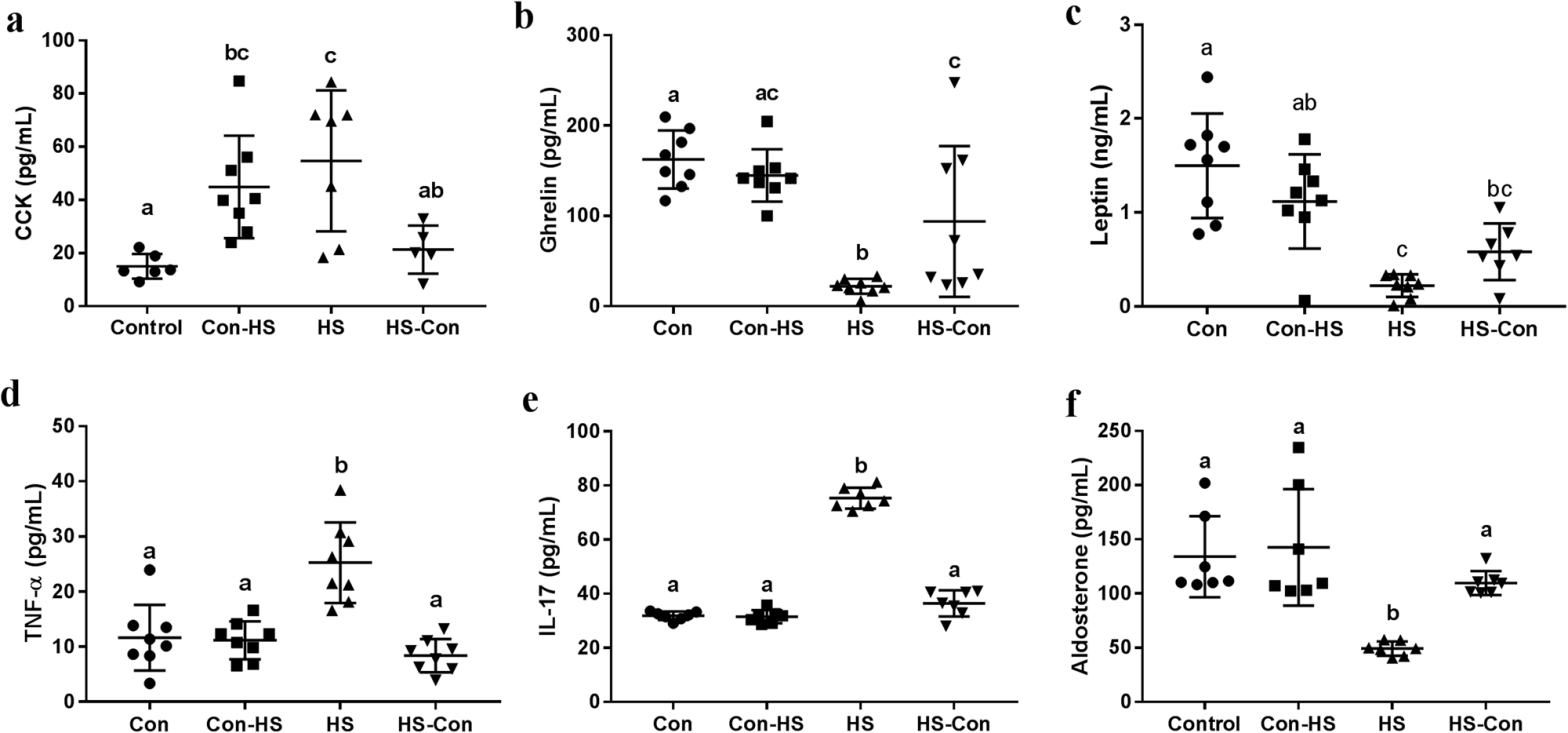
Microbiota transplant regulated gastrointestinal hormones and alleviated systemic inflammation. The concentrations of cholecystokinin (CCK, *P* = 0.002) (**a**), des-acylated ghrelin (*P* < 0.001) (**b**), leptin (*P* < 0.001) (**c**), tumor necrosis factor (TNF-α, *P* < 0.001) (**d**), interleukin 17 (IL-17, *P* < 0.001) (**e**) and aldosterone (*P* < 0.001) (**f**). Data are presented as means ± standard error of the mean (s.e.m), and bars which do not share the same letter are significantly different from each other (*P* < 0.05). Con, gerbils drank tap water ad libitum and received sterile saline as sham-CMT via oral gavage; Con-HS, Con gerbils received microbiota from HS gerbils via oral gavage; HS, gerbils drank water with 8% NaCl ad libitum and received sterile saline as sham-CMT via oral gavage; HS-Con, gerbils drank HS water ad libitum and received microbiota from the Con gerbils via oral gavage.

To determine whether HS intake and gut microbiota affect gastrointestinal hormones and inflammatory responses, we measured concentrations of circulating CCK (a satiety signal), ghrelin (an orexigenic signal) and leptin (one of adipocytokines), and proinflammatory cytokines (which are essential for host defense against pathogenic bacteria and fungi) including tumor necrosis factor α (TNF-α) and interleukin 17 (IL-17) at the end of the 20-week period. Concomitant with the lowered body mass and food intake, HS gerbils displayed higher serum concentrations of CCK (ANOVA, *F*_3,22_ = 6.846, *P* = 0.002; Fig. 3a), and lower concentrations of des-acylated ghrelin (ANOVA, *F*_3,28_ = 14.190, *P* < 0.001; Fig. 3b) and leptin than Con gerbils (ANOVA, *F*_3,28_ = 14.679, *P* < 0.001; Fig. 3c). However, CMT reduced these changes in hormone concentrations in HS-Con. The HS group had a 1.2 fold higher serum concentration of TNF-α (ANOVA, *F*_3,28_ = 16.626, *P* < 0.001; Fig. 3d) and a 1.4 fold higher serum concentration of IL-17 (ANOVA, *F*_3,24_ = 269.376, *P* < 0.001; Fig. 3e) than the Con group. Consistent with the known role of aldosterone in sodium/water balance, HS gerbils reduced serum aldosterone concentration to reduce sodium reuptake compared with Con gerbils (ANOVA, *F*_3,28_ = 11.171, *P* < 0.001; Fig. 3f). However, CMT from Con donors to HS recipients alleviated HS-induced changes in circulating hormones and inflammation (Fig. 3).

### Gut microbiota regulated the expression of intestinal and renal AQP2 and α-ENaC

**Fig. 4:**
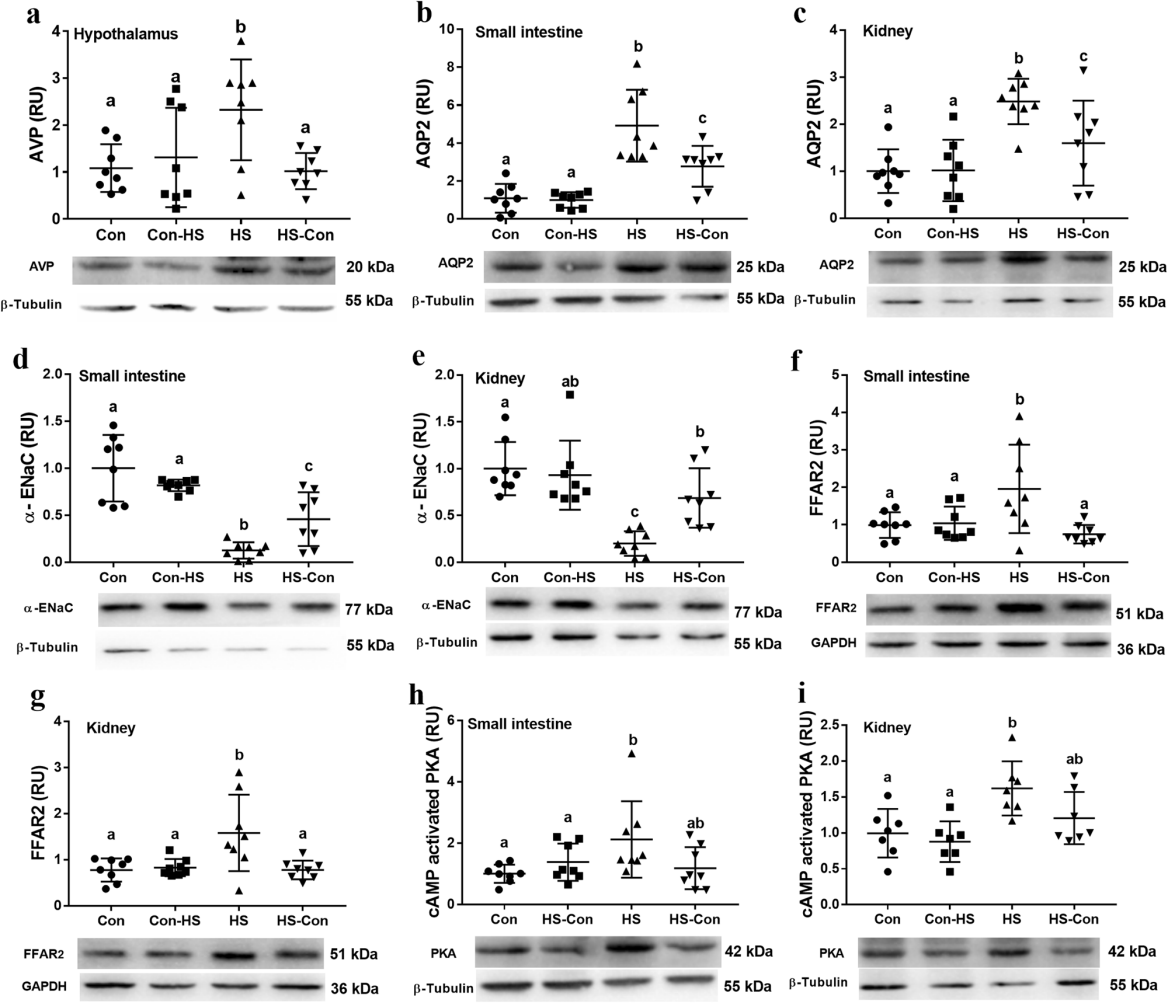
Caecal microbiota transplant (CMT) affected molecular markers related to osmoregulation. Arginine vasopressin (AVP) in the hypothalamus (*P* = 0.012) (**a**); aquaporin 2 (AQP2) in small intestine (*P* < 0.001) (**b**) and in kidney (*P* < 0.001) (**c**); epithelial sodium channel (α-ENaC) in small intestine (*P* < 0.001) (**d**) and in kidney (*P* < 0.001) (**e**); free fatty acid receptor 2 (FFAR2) in small intestine (*P* = 0.006) (**f**) and in kidney (*P* = 0.003) (**g**); and cAMP-activated Protein Kinase (PKA) in small intestine (*P* = 0.026) (**h**) and in kidney (*P* < 0.001) (**i**). All blots derived from the same experiment and were processed in parallel. Data are presented as means ± standard error of the mean (s.e.m), and bars which do not share the same letter are significantly different from each other (*P* < 0.05). Con, gerbils drank tap water ad libitum and received sterile saline as sham-CMT via oral gavage; Con-HS, Con gerbils received microbiota from HS gerbils via oral gavage; HS, gerbils drank water with 8% NaCl ad libitum and received sterile saline as sham-CMT via oral gavage; HS-Con, gerbils drank HS water ad libitum and received microbiota from the Con gerbils via oral gavage.

At the end of the 20-week period, proteins known to play a role in salt/water balance, including hypothalamic AVP, and intestinal and renal AQP2 and α-ENaC, in signaling pathway (PKA pathway), and in gut microbial signals such as the receptor FFAR2 of SCFAs were measured. The AVP expression in the hypothalamus (ANOVA, *F*_3,28_ = 4.407, *P* = 0.012; Fig. 4a), and AQP2 expressions in the small intestine (ANOVA, *F*_3,28_ = 18.258, *P* < 0.001; Fig. 4b) and kidney (ANOVA, *F*_3,28_ = 19.733, *P* < 0.001; Fig. 4c) were greater in HS than Con gerbils, which is consistent with the known roles of AVP and AQP2 to support water retention. However, this change was reduced in the HS-Con group. The α-ENaC expressions in the small intestine (ANOVA, *F*_3,28_ = 13.781, *P* < 0.001; Fig. 4d) and kidney (ANOVA, *F*_3,28_ = 21.979, *P* < 0.001; Fig. 3e) were lower in the HS than the Con gerbils, enabling the HS gerbils to promote sodium excretion. The CMT in the HS gerbils from Con donors buffered this regulation. The expressions of FFAR2 and cAMP-activated PKA both in the small intestine and kidney were greater in the HS than Con group (*P* < 0.01; Fig. 4f–i), and these changes were attenuated in the HS-Con gerbils.

### Microbiota transplant restrucured faecal microbiota community

**Fig. 5:**
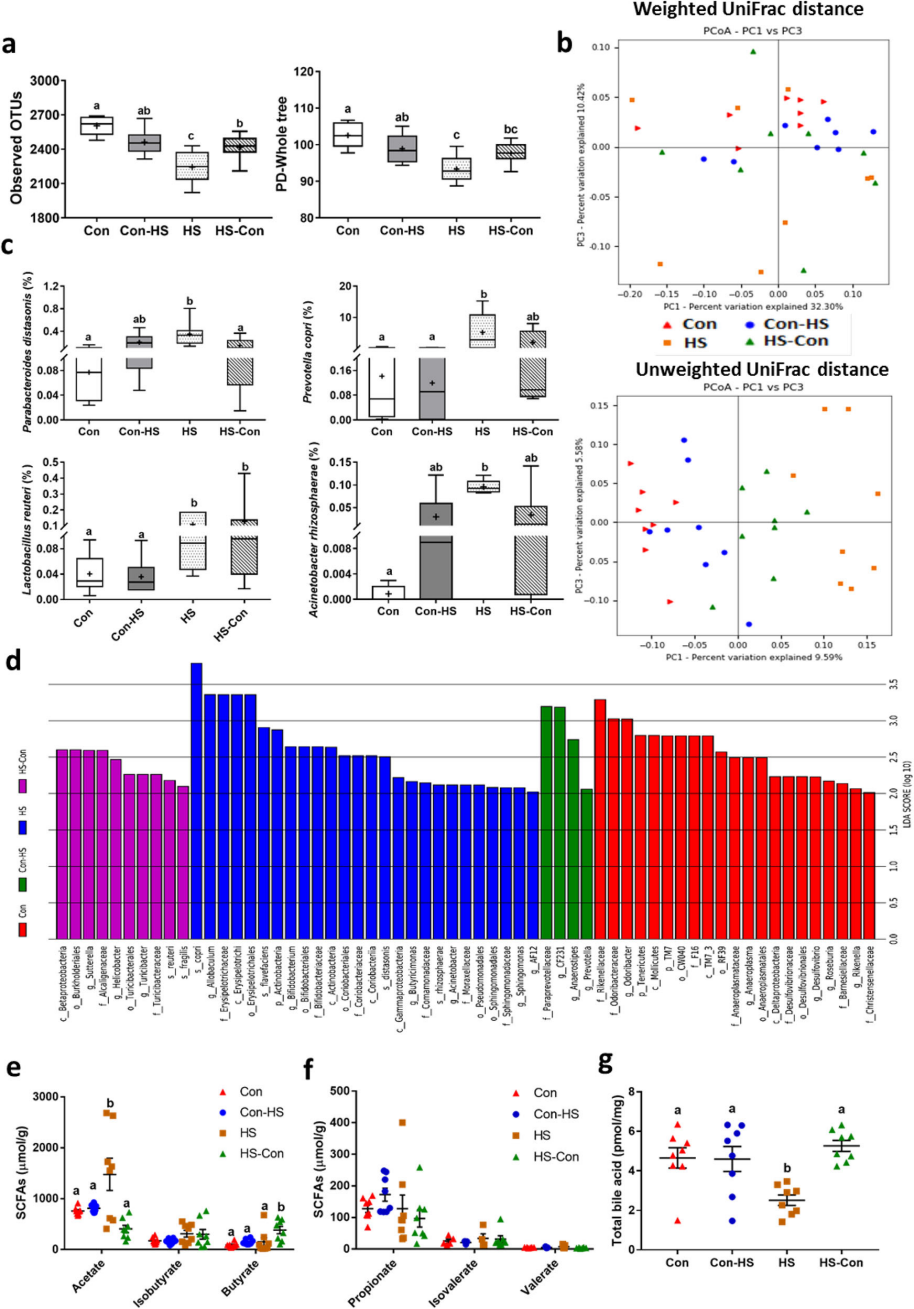
Caecal microbiota transplant (CMT) restructured the gut microbiota profile. Observed operation taxonomic units (OTUs, *P* < 0.001), and phylogenetic diversity (PD) whole tree (*P* < 0.001) (**a**); principal coordinate analyses (PCoA) plots based on weighted (*P* = 0.004) and unweighted (*P* = 0.001) UniFrac distances in the faecal microbiota of different groups (**b**); relative abundances of different bacteria at the species level in the experimental groups (**c**); differential bacterial taxa selected by LEfSe analysis with LDA > 2 in the faecal bacterial community (**d**); faecal concentrations of different short-chain fatty acids (SCFAs) (**e**, **f**) and serum concentrations of total bile acids (**g**). Data are presented as means ± standard error of the mean (s.e.m), and bars which do not share the same letter are significantly different from each other (*P* < 0.05). Con, gerbils drank tap water ad libitum and received sterile saline as sham-CMT via oral gavage; Con-HS, Con gerbils received microbiota from HS gerbils via oral gavage; HS, gerbils drank water with 8% NaCl ad libitum and received sterile saline as sham-CMT via oral gavage; HS-Con, gerbils drank HS water ad libitum and received microbiota from the Con gerbils via oral gavage.

We further determined whether transplant of normal microbiota restructured the gut microbiota profile and reshaped the bacterial metabolites. Two weeks after CMT (in week 6), α-diversity was lower in HS than Con gerbils (observed OTUs, *F*_3_, _28_ = 14.724, *P* < 0.001; PD whole tree, *F*_3_,_28_ = 10.065, *P* < 0.001; Fig. 5a and Supplementary Table 2), but the HS-Con group did not differ from the Con group (*post hoc*, *P* > 0.05; Fig. 5a). The β-diversity displayed a clear separation between the HS group and the other groups (ANOSIM, weighted, *R* = 0.134, *P* = 0.004; unweighted, *R* = 0.509, *P* = 0.001; Fig. 5b). At the species level, the relative abundances of *Parabacteroides distasonis* (*F*_3,28_ = 5.471, *P* = 0.004), *Prevotella copri* (*F*_3,28_ = 3.914, *P* = 0.019), and *Lactobacillus reuteri* (*F*_3,28_ = 2.986, *P* = 0.048) were higher in HS than Con group (Fig. 5c), and in the HS-Con, except for *L*. *reuteri*, these bacterial species recovered to control levels (*P* > 0.05; Fig. 5c). The distinctive bacteria in each group were identified by the LEfSe method (Fig. 5d). Acetate concentration was higher in the HS than Con group (ANOVA, *F*_3,28_ = 7.582, *P* = 0.001; Fig. 5e); however, CMT (HS-Con) decreased acetate but increased butyrate concentration (ANOVA, *F*_3,28_ = 5.509, *P* = 0.004; Fig. 5e). Other SCFA concentrations were not affected by salt intake or CMT (Fig. 5e, f). Since the gut microbiota are also involved in host bile acid metabolism, we measured serum total bile acids and observed a lower concentration in HS than Con gerbils (ANOVA, *F*_3, 28_ = 7.047, *P* < 0.001; Fig. 5g). With CMT, however, the concentration of the total bile acids was recovered.

Even 16 weeks after CMT (in week 20), the α diversity of microbial community was still lower in HS than Con gerbils (observed OTUs, *F*_3,24_ = 5.336, *P* = 0.006; PD whole tree, *F*_3,24_ = 4.642, *P* = 0.011; Supplementary Fig. 2a and Supplementary Table 3); however, it recovered to the Con level in the HS-Con group (post hoc, *P* > 0.05). The β diversity displayed separation between the HS and other groups (ANOSIM, weighted, *R* = 0.146, *P* = 0.009; unweighted, *R* = 0.424, *P* = 0.001; Supplementary Fig. 2b), and distinctive biomarkers of bacteria were identified in each group (Supplementary Fig. 2c).

## Discussion

Desert mammals must balance water and electrolytes to survive in their habitats. The present study showed that salt intake altered the gut microbial community, accompanied by decreased relative abundances of *Rikenella* and *Christensenella* but increased relative abundance of *Atopobium*. The HS gerbils concentrated urine highly by increasing hypothalamic AVP and intestinal and renal AQP2 to support water retention, and reduced intestinal and renal α-ENaC to promote sodium excretion. These responses resulted in negative energy balance and induced systemic inflammation in the gerbils. The CMT from Con donors to HS recipients reversed these responses, and maintained osmoregulation by interaction with the host’s intestine and kidney. These data indicate that the microbiota-gut-kidney axis is involved in mediating osmoregulation in desert mammals.

### Desert mammals can concentrate urine in response to salt intake and water shortage

Desert mammals have the ability to produce highly hyperosmotic urine, which is a vital adaptation that enables them to survive in deserts with little water and high salt intakes^22^. Our studies showed that Mongolian gerbils increased urine osmolality but decreased serum osmolality to meet high salt challenge, which was also observed in the previous study^11^. In contrast, laboratory mice and spiny mice (*Acomys cahirinus*) decreased urine osmolality but increased plasma osmolality in response to a high-salt diet^23^. The HS gerbils were able to survive for >20 weeks with high urine osmolality, accompanied by reduced food intake and a low set-point of body mass. The reductions in anorexigenic CCK and orexigenic ghrelin levels depressed appetite (16–18), and the drop in serum leptin, which is secreted by white adipocytes and gastric epithelial cells^24,25^, was concomitant with the loss in body mass in HS gerbils. With salt loading, the gerbils relied on AVP-dependent AQP2 activation to promote water reabsorption both in the small intestine and kidney, unlike reports for rats and humans in which water transport via AQP2 increased by an AVP-independent mechanism^26^. In addition, these gerbils decreased serum aldosterone, and reduced intestinal and renal α-ENaC expression to restrain sodium reabsorption^11^. These data indicate that Mongolian gerbils are much more tolerant and better adapted than humans and mice in handling high sodium intake, in particular in their ability to concentrate urine.

### High salt consumption disturbed the gut microbiota community

Salt consumption depressed the diversity of the gut microbiota community and altered microbial composition. The relative abundance of several intestinal bacteria, particularly *Parabacteroides distasonis* and *Prevotella copri*, increased with high salt intake. It was reported that *Parabacteroides distasonis* altered the bile acid profile with elevated lithocholic acid and ursodeoxycholic acid, increased succinate concentration in the gut and caused a loss in body mass^27^. A HS diet increased the relative abundance of *Prevotella* spp. in the gut of mice and humans, and these bacteria have been associated with diseases such as systemic autoimmunity and inflammation^28,29^. Similar with other species on a high salt diet^30^, the HS gerbils displayed systemic inflammation, which is consistent with the changes in gut bacteria. Moreover, *Prevotella copri* has been linked to high digestibility^31^, and consequently, the increased abundance of *Prevotella copri* in the HS gerbils can be of importance for survival when food intake is reduced.

### Microbiota transplant rescued the gut microbiota and alleviated host osmoregulation

Transplanting bacteria from Con to HS gerbils led to the restructuring and recovery of the microbial community. This was accomplished by reducing the relative abundances of pathogenic bacteria such as *Prevotella copri* and *Parabacteroides distasonis*, and increasing the relative abundance of beneficial bacteria such as *Lactobacillus reuteri* in HS–Con when compared to HS. It was reported that *Prevotella copri* was correlated negatively with body mass^32^, and *Parabacteroides distasonis* prevented body mass gain of the host^27^. Studies demonstrated that treatment of HS mice with *Lactobacillus murinus* prevented salt-induced autoimmune encephalomyelitis^30^. These data suggest that CMT-induced reshaping of the gut microbiota may be linked to reduced systemic inflammation and the recovery in energy balance. Consistent with the recovery in food intake, the circulating CCK, ghrelin and leptin concentrations recovered to the Con levels, supporting that the gut microbiota can regulate the secretion of gastrointestinal hormones and adipocytokines. However, the CMT from HS to Con gerbils did not alter the gut microbiota and host physiological phenotypes, indicating that the bacteria from a community with low diversity and richness failed to colonize in a healthy recipient’s gut. This may be a consequence of community competition and function saturation^33^.

In addition to the recovery of the gut microbial community, CMT from Con donors to HS gerbils increased the concentration of total bile acids and decreased the concentration of acetate, but increased the concentration of butyrate, which may be due to the high relative abundance of *Lactobacillus reuteri*, an important contributor to butyrate production^34^. In vitro and in vivo studies demonstrated that butyrate can stimulate anti-diuretic hormone (ADH) secretion, and electro-neutral NaCl absorption from the colon through induction of Na^+^/H^+^-exchangers, whereas acetate had little effects on ADH secretion and urine osmolality^16,35,36^. Moreover, in previous studies, butyrate induced intestinal ENaC expression and regulated electrogenic sodium absorption^37,38^. Therefore, the increase in butyrate can explain, at least in part, why urine osmolality was not completely restored by CMT from Con donors to HS recipients. In addition, the CMT-induced increase in butyrate, together with the activation of cAMP-PKA-CREB signaling pathway, promoted renal AQP2 expression to increase water permeability^39^. These data indicate that gut microbiota, via SCFAs, are involved in osmoregulation of salt-loaded Mongolian gerbils.

**Fig. 6:**
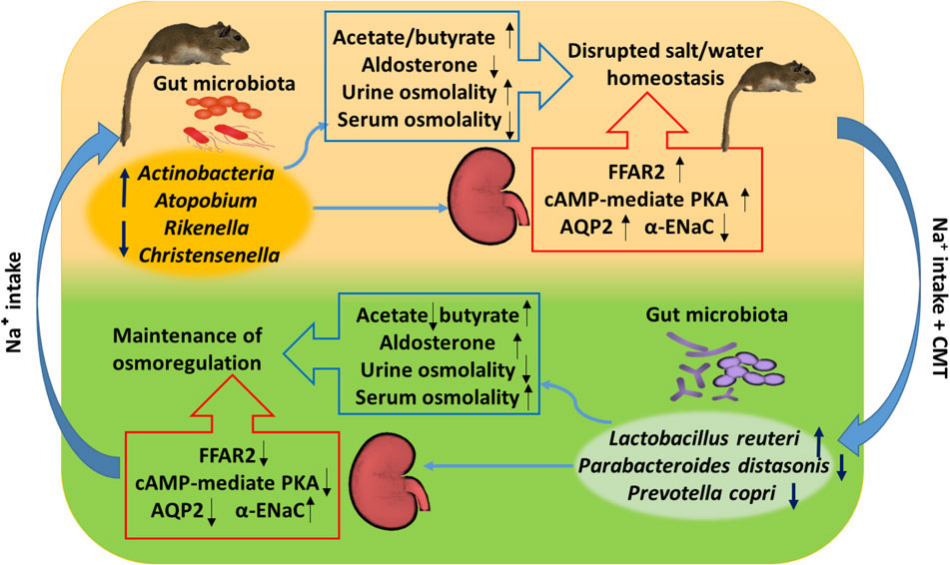
The paradigm summarizing the microbiota-gut-kidney axis in mediating salt-related osmoregulation. Salt intake reduced the gut microbiota diversity and disrupted salt/water homeostasis. Caecal microbiota transplant (CMT) from the control to salt-treated gerbils improved the gut microbiota community with enriched beneficial bacteria such as *Lactobacillus reuteri*, but decreased potential pathogenic bacteria such as *Parabacteroides distasonis* and *Prevotella copri*. The recovery in gut bacteria and increase in short chain fatty acids, particularly butyrate, after CMT was linked with alterations in intestinal and renal proteins, including aquaporin 2 (AQP2), epithelial sodium channel α (α-ENaC), free fatty acid receptor 2 (FFAR2) and cAMP–PKA, which play roles in osmoregulation and microbial signals. This image and every element of this image were created by the authors and no any previously-created elements were used in this image.

In summary, the findings provide evidence that the microbiota-gut-kidney axis was involved in mediating salt-related osmoregulation. Salt intake altered the gut microbiota and the concentration of short chain fatty acids SCFAs, which led to negative energy balance and systemic inflammation. With high salt loading, the gerbils enhanced AQP2 and decreased α-ENaC expression both in the small intestine and kidney to promote water retention and sodium excretion. Transplantation of gut microbiota from Con donors to HS gerbils restructured the gut microbiota community, by reducing the relative abundances of *Parabacteroides distasonis* and *Prevotella copri*, but increasing relative abundance of *Lactobacillus reuteri*. The restructuring of the gut bacteria by CMT was followed by changes in microbial metabolites and proteins known to play a role in salt/water balance, leading to maintenance of osmoregulation (Fig. 6). These findings demonstrate a link among the microbiota, gut and kidney in regulating sodium and water balances for desert rodents to survive in the salt-loading habitat. Further studies are still required to elucidate the underlying mechanisms by which the gut microbiota and SCFAs regulate salt/water metabolism and osmolality in Mongolian gerbils.

## Methods

### Animals and housing

Female Mongolian gerbils, aged 6 to 12 months, were housed individually in plastic cages (30 × 15 × 20 cm) at the Institute of Zoology, the Chinese Academy of Sciences (CAS). The animals were maintained on sawdust bedding under a 16 L: 8D photoperiod regime and an air temperature of 23 ± 1 °C. The gerbils were fed a standard rodent pellet chow (Beijing KeAo Bioscience Co) and were offered water ad libitum. The protocol and procedures in this study were approved by the Animal Care and Use Committee of the Institute of Zoology, CAS.

### Experimental designs

Experiment 1 examined the effect of NaCl intake on diversity and relative abundance of the gut microbial community. A total of 30 adult gerbils were divided randomly into 3 groups, (*n* = 10 per group), each receiving drinking water ad libitum with a different concentration of NaCl: (1) controls (Con), with no added NaCl; (2) medium salt (MS) with 4% NaCl; and, (3) high salt (HS) with 8% NaCl for 4 weeks. For collection of faecal samples, each gerbil was moved into a clean cage without bedding after 4 weeks. Fresh faecal pellets were collected immediately in sterilized tubes after defecation, snap-frozen in liquid nitrogen and stored at −80 °C for later measurements of gut microbiota and SCFAs.

Experiment 2 examined the role of gut microbiota in mediating host salt and water balances using high-salt intake as a model. Another set of 32 Mongolian gerbils at age of 6 months were divided randomly into two groups: one HS group receiving drinking water with 8% NaCl ad libitum (*n* = 16) and one Con group with no added NaCl (*n* = 16). After 4 weeks, the Con gerbils were gavaged with either caecal microbiota from the HS group (Con-HS; *n* = 8) or with sterile saline (Con; *n* = 8), and the HS gerbils were gavaged either with caecal microbiota from the Con group (HS-Con; *n* = 8) or with sterile saline (HS; *n* = 8) and continued receiving drinking water with 8% NaCl during the whole acclimation period (total 20 weeks, Fig. 2a). Body mass (±0.1 g), food intake and water intake were measured at 09:00 a.m. every 4 weeks. Samples of urine, faeces and blood were collected prior to acclimation and in the 4th, 6th and 20th weeks of acclimation. Urine samples were collected immediately after urination for measurements of osmolality. Fresh faecal pellets were collected as described in experiment 1 in the 6th (2 weeks after CMT) and 20th weeks (16 weeks after CMT) of acclimation. The blood was collected from the infraorbital vein. The gerbils were sacrificed by CO_2_ asphyxiation between 09:00 and 11:00 at the end of the 20-week period. Blood samples were collected from the carotid artery and centrifuged at 1500 g for 30 min to obtain serum. The hypothalamus, kidneys and the digestive tract (stomach, small intestine and cecum) were excised, snap-frozen in liquid nitrogen and then stored at −80 °C for later measurements.

### Caecal microbiota transplant (CMT)

Caecal contents were collected and combined from 3 donor gerbils of control and 3 donor gerbils of the HS group. The contents (200 mg) were dissolved in 2 mL sterile saline (0.9% sodium chloride), and then a 200 μL suspension was delivered via oral gavage to the recipient gerbils for 3 days (once daily) as described previously^12^. For sham CMT, the recipient gerbils received oral gavages of 200 μL sterile saline to match the stress of gavage manipulation during the same 3 days.

### Measurements of serum and urine osmolalities and serum hormones

Serum and urine osmolalities were measured in duplicate using an osmometer (SMC 30 B, Tianhe Medical Instruments, Tianjin, China).

Serum concentrations of total bile acids were measured using the Total Bile Acids Assay Kit (Colorimetric) (Catalog No. ab239702, Abcam, Cambridge MA, USA). According to the manufacturer’s instructions, the standards with different volumes and the serum (25 μL) were added to a 96-well plate and were adjusted to 50 μL with ddH_2_O. Then, 100 μL Probe mix was added to the wells of each sample and standard, and the plate was incubated for 10 min at 37 ^o^C. Finally, 50 μL of the reaction mixture was added to the wells and absorbance was measured at 405 nm in a kinetic mode at 37 °C for 60 min and protected from light.

Serum CCK (Catalog No. CEA802Mu, Cloud-Clone Corp., Wuhan, China), ghrelin (Catalog No. CEA991Ra) and leptin (Catalog No. SEA084Ra) were determined by ELISA kits according to the manufacturer’s instruction. The minimum detectable concentrations were 4.61 pg/mL for CCK, 52.3 pg/mL for ghrelin and 0.129 ng/mL for leptin. Serum TNF-α was quantified with an enzyme-linked immunosorbent assay using an ELISA kit (Catalog No. SEA133Hu). Serum IL-17 concentration was determined using ELISA kit (Catalog No. SEA063Mu). The lower limits of detection were 6.5 pg/mL for TNF-α and 5.6 pg/mL for IL-17. Serum concentration of aldosterone was determined using an Aldosterone EIA Kit (Catalog No. ADI-900-173, Enzo Life Science, NY, USA). The minimum detectable concentration was 4.7 pg/mL.

### Measurements of proteins related with osmoregulation and microbial signals

The total protein concentrations of the hypothalamus, small intestine segments and kidney samples were measured by the Folin phenol method using bovine serum albumin as a standard. The proteins of AVP in the hypothalamus, and AQP2, α-ENaC, FFAR2 and cAMP-activated PKA in small intestine and kidney were measured by Western blot as described previously^10,11,40^. Total proteins of tissues were separated in discontinuous 10% or 12% SDS-polyacrylamide gels. The protein marker (20350ES90; Yeasen, Shanghai, China) covering the expected molecular weight range was loaded to the wells on both sides for later accurately cutting of the gels and estimation of the detected proteins. The proteins and marker were then transferred onto polyvinylidene difluoride (PVDF) membranes (Hybond-P; Amersham, Buckinghamshire, UK). The membranes were blocked in 5% non-fat dry milk for 2 h at room temperature and then incubated in a plastic box at 4 °C overnight with anti-AVP antibody (AB1565; Merck Millipore, Darmstadt, Germany), anti-AQP2 antibody (sc-9882; Santa Cruz Biotechnology, CA, USA), anti-α-ENaC antibody (PA1-920A; Thermo Fisher, Waltham, MA, USA), anti-FFAR2/GPR43 antibody (ABC299; Merck Millipore, Darmstadt, Germany), anti-cAMP protein kinase catalytic subunit antibody (ab26322; Abcam, Cambridge, UK), anti-β-Tubulin antibody (30301ES60; Yeasen, Shanghai, China) and anti-GAPDH antibody (30201ES60; Yeasen)^10,11,40^. The immunoblot was visualized with horseradish peroxidase-conjugated secondary antibodies that were either goat anti-rabbit IgG (11-035-003; Jackson), goat anti-mouse IgG (33201ES60; Yeasen) or rabbit anti-goat IgG (ZB-2306; Zsbio). Protein blots were detected with chemoluminescence (ECL, Amersham Life Sciences, Little Chalfont, UK) and quantified using Quantity One software (version 4.4.0, BioRad, Hercules, CA, USA). The protein content was expressed as relative units (RU). All blots derived from the same experiment and were processed in parallel. The uncropped and unprocessed scans of the most important blots were supplied in the supplementary information (Supplementary Fig. 3).

### Short chain fatty acids (SCFAs)

SCFAs were extracted from fresh faecal pellets (0.2 g) using ddH_2_O (200 mL) and centrifuged at 16,200 g at 4 °C for 20 min. H_3_PO_4_ (25%, 6 μL) was added to the supernatant (54 μL) at a ratio of 1:9 and the supernatant was then filtered through a centrifugal filter (0.22 μm). Six SCFAs, including acetate, propionate, butyrate, isobutyrate, valerate, and isovalerate, were identified and quantified by gas chromatography (GC, Agilent7890A; Agilent Technologies) with a GC autosampler and an FID system^12^. The GC was equipped with a DB-WAX column (Polyethylene Glycol 20000, 30 m × 0.25 mm [ID], film thickness 0.25 µm, Agilent Technologies, Palo Alto, CA, USA).

### DNA extraction

Total faecal DNA was extracted using 2 × CTAB (cetyltrimethyl ammonium bromide), phenol chloroform mixture (phenol: chloroform: isoamyl alcohol = 25:24:1) and was isolated via the spin column from SanPrep Column DNA Gel Extraction Kit (Sangon Biotech, Shanghai, China). DNA purity was assessed by absorbance on a Nanodrop 2000 (Thermo Fisher Scientific, Carlsbad, CA, USA) by measuring the A260/A280 ratio.

### 16 S rRNA gene amplicon sequencing and analysis

The V3–V4 hypervariable regions of the 16 S rRNA gene were amplified using two universal primers (forward primer-341F, CCTACGGGNGGCWGCAG; reverse primer-805R, GACTACHVGGGTATCTAATCC^12,40^. The PCR reaction (total 20 μL) was prepared as follows: template DNA 2 μL, amplicon PCR forward primer (10 μM) 1 μL, amplicon PCR reverse primer (10 μM) 1 μL, and 2×Taq PCR MasterMix 16 μL. PCR was performed in the same thermal cycler (SimpliAmp, ABI) using the following program: 1 cycle of denaturing at 94 °C for 3 min, 6 cycles of denaturing at 94 °C for 20 s, annealing at 55 °C for 30 s, elongation at 72 °C for 30 s, and followed by 30 cycles of denaturing at 94 °C for 15 s, annealing at 68 °C for 15 s, elongation at 72 °C for 20 s, and a final extension at 72 °C for 5 min. The PCR products were detected using agarose gel (1%, w/v) electrophoresis stained with ethidium bromide and visualized under UV light. PCR products were purified using SanPrep Column DNA Gel Extraction Kit (GE0101-200, TsingKe, Beijing, China), according to the manufacturer’s instructions. The sequencing was done on an Illumina HiSeq 2500.

The 16 S sequence paired-end data set was joined and quality was filtered using the FLASH method^41^. All sequences analysis used QIIME (version 1.9.1) software suite, according to the Qiime tutorial (http://qiime.org/) with some modifications^42^. Chimeric sequences were removed using usearch61 with de novo models^43^. Sequences were clustered against the 2013 Greengenes (13_8 release) ribosomal database’s 97% reference dataset. Sequences that did not match any entries in this reference were subsequently clustered into de novo OTUs at 97% similarity with UCLUST. Taxonomy was assigned to all OTUs using the RDP classifier within QIIME and the Greengenes reference data set. Rarefaction and rank abundance curves were calculated from OTU tables using alpha diversity and rank abundance scripts within the QIIME pipeline. The hierarchical clustering, based on population profiles of most common and abundant taxa, was performed through UPGMA clustering (unweighted pair group method with arithmetic mean, also known as average linkage) on the distance matrix of OTU abundance and a Newick formatted tree was obtained using the QIIME package.

### Statistical analyses

All analyses used SPSS 20.0 (SPSS Inc., Chicago, IL, USA). Body mass was analyzed by repeated measures ANOVA followed by Tukey’s post hoc tests and food and water intakes were analyzed among groups by repeated measures ANCOVA (with body mass as a covariate) followed by Tukey’s *post hoc* tests. All other parameters were analyzed by one-way ANOVA and group differences were separated by Tukey’s post hoc tests. Results are presented as means ± standard error of the mean (s.e.m), and the statistical significance was accepted at *P* < 0.05.

Alpha diversity was used as a measure of richness and diversity of bacteria. Differences in relative abundance of bacteria among treatments were tested by one-way ANOVA. The β diversity (PCoA) was analyzed based on unweighted and weighted UniFrac distances using evenly sampled OTU abundances, and statistical significances among groups were analyzed by ANOSIM by 999 permutations. The LEfSe method was used to assess differences in microbial communities using an LDA score threshold of 2^44^. The graphics were developed in STAMPv2.1.3. (http://kiwi.cs.dal.ca/Software/STAMP)^45^. QIIME and R codes were supplied as supplementary information. The level of statistical significance was set at *P* < 0.05 (False Discovery Rate (FDR)-corrected).

### Reporting summary

Further information on research design is available in the Nature Research Reporting Summary linked to this article.

## Supplementary information

Supplementary file

Reporting summary

Resupplied SI file for production

## Data Availability

The raw data of 16 S rRNA gene amplicon sequence are available in the NCBI Sequence Read Archive under accession PRJNA759876 and PRJNA796757.

## Code availability

QIIME and R codes that were used in the 16 S rRNA gene amplicon sequencing and analysis are available in the supplementary file.
