# Supplementary material for: The microbiota-gut-kidney axis mediates host osmoregulation in a small desert mammal
Source: NPJ Biofilms Microbiomes. 2022 Apr 4;8:16. doi: 10.1038/s41522-022-00280-5 (PMC8980004; doi:10.1038/s41522-022-00280-5)
Supplement: Supplementary file 1 — Supplementary file [file 41522_2022_280_MOESM1_ESM.pdf]

The microbiota-gut-kidney axis mediates host osmoregulation in a small desert mammal

Zahra Nouri, Xue-Ying Zhang, Saeid Khakisahneh, Abraham Allan Degen, De-Hua Wang

Supplementary Table 1 Salt intake induced a reduction in α diversity of faecal microbiota community in Experiment 1.

	Chao1	Observed OTUs	Shannon	Simpson	PD whole tree
Con	10199 ± 217.2[a]	3622 ± 76.3 [a]	9.04 ± 0.14[a]	0.99±0.002	151.8 ± 2.10 [a]
MS	9762 ± 211.5 [ab]	3444 ± 67.0 [ab]	8.76 ± 0.14[ab]	0.98±0.003	145.9 ± 2.00 [ab]
HS	8712 ± 464.0 [b]	3120 ± 139.22 [b]	8.48 ± 0.14[b]	0.98±0.002	137.6 ± 4.61 [b]
F	5.70	6.53	4.01	1.092	5.15
P	0.009	0.005	0.030	0.350	0.013

Data are presented as means ± standard error of the mean (s.e.m), and columns which do not share the same letter are significantly different from each other ($P < 0.05$). Con, Control (no added NaCl in drinking water); MS, moderate salt (4% NaCl in drinking water); HS, high salt (8% NaCl in drinking water).

Supplementary Table 2. The α diversity of faecal microbiota community 2 weeks after caecal microbiota transplant (CMT) in Experiment 2.

	Chao1	Observed OTUs	Shannon	Simpson	PD whole tree
Con	3731 ± 29.4[a]	2605 ±27.2 [a]	8.66 ±0.06 [a]	0.99 ±0.001[a]	102.6 ±1.19[a]
Con-HS	3602 ±49.4[ab]	2461 ±38.4 [ab]	8.23 ±0.08 [b]	0.98 ±a0.001[b]	98.9 ±1.34 [ab]
HS	3315 ±55.2 [c]	2244 ±48.9 [c]	8.03 ±0.08 [b]	0.98 ±0.002[b]	93.4 ±1.24 [c]
HS-Con	3534 ±41.3[b]	2420 ±37.5[b]	8.31 ±0.10 [b]	0.99 ±0.001[ab]	97.7 ±0.98[bc]
F	14.94	14.72	10.75	3.836	10.06
P	< 0.001	< 0.001	< 0.001	0.020	< 0.001

Data are presented as means ± standard error of the mean (s.e.m), and columns which do not share the same letter are significantly different from each other ($P < 0.05$). Con, gerbils drank tap water *ad libitum* and received sterile saline as sham-CMT via oral gavage; Con-HS, Con gerbils received microbiota from HS gerbils via oral gavage; HS, gerbils drank water with 8% NaCl *ad libitum* and

received sterile saline as sham-CMT via oral gavage; HS-Con, gerbils drank HS water *ad libitum* and received microbiota from the Con gerbils via oral gavage.

Supplementary Table 3. The α diversity of faecal microbiota community 16 weeks after caecal microbiota transfer (CMT) in Experiment 2.

	Chao1	Observed OTUs	Shannon	Simpson	PD whole tree
Con	4119 ± 72.3	2721 ±33.1^{a}	9.00 ±0.04^{a}	0.99 ±0.001	124.7 ±1.29^{a}
Con-HS	4131 ±85.7	2729 ±31.2^{a}	8.86 ±0.07^{a}	0.99 ±a0.001	123.3 ±1.15^{a}
HS	3905 ±66.8	2483 ±46.6^{b}	8.45 ±0.10 ^{b}	0.98 ±0.002	116.6 ±0.92^{b}
HS-Con	3995 ±123.4	2539 ±87.4^{ab}	8.67 ±0.14^{ab}	0.99 ±0.005	119.9 ±2.72^{ab}
F	1.434	5.336	6.219	2.278	4.642
P	0.257	0.006	0.003	0.105	0.011

Data are presented as means ± standard error of the mean (s.e.m), and columns which do not share the same letter are significantly different from each other ($P < 0.05$). Con, gerbils drank tap water *ad libitum* and received sterile saline as sham-CMT via oral gavage; Con-HS, Con gerbils received microbiota from HS gerbils via oral gavage; HS, gerbils drank water with 8% NaCl *ad libitum* and received sterile saline as sham-CMT via oral gavage; HS-Con, gerbils drank HS water *ad libitum* and received microbiota from the Con gerbils via oral gavage.

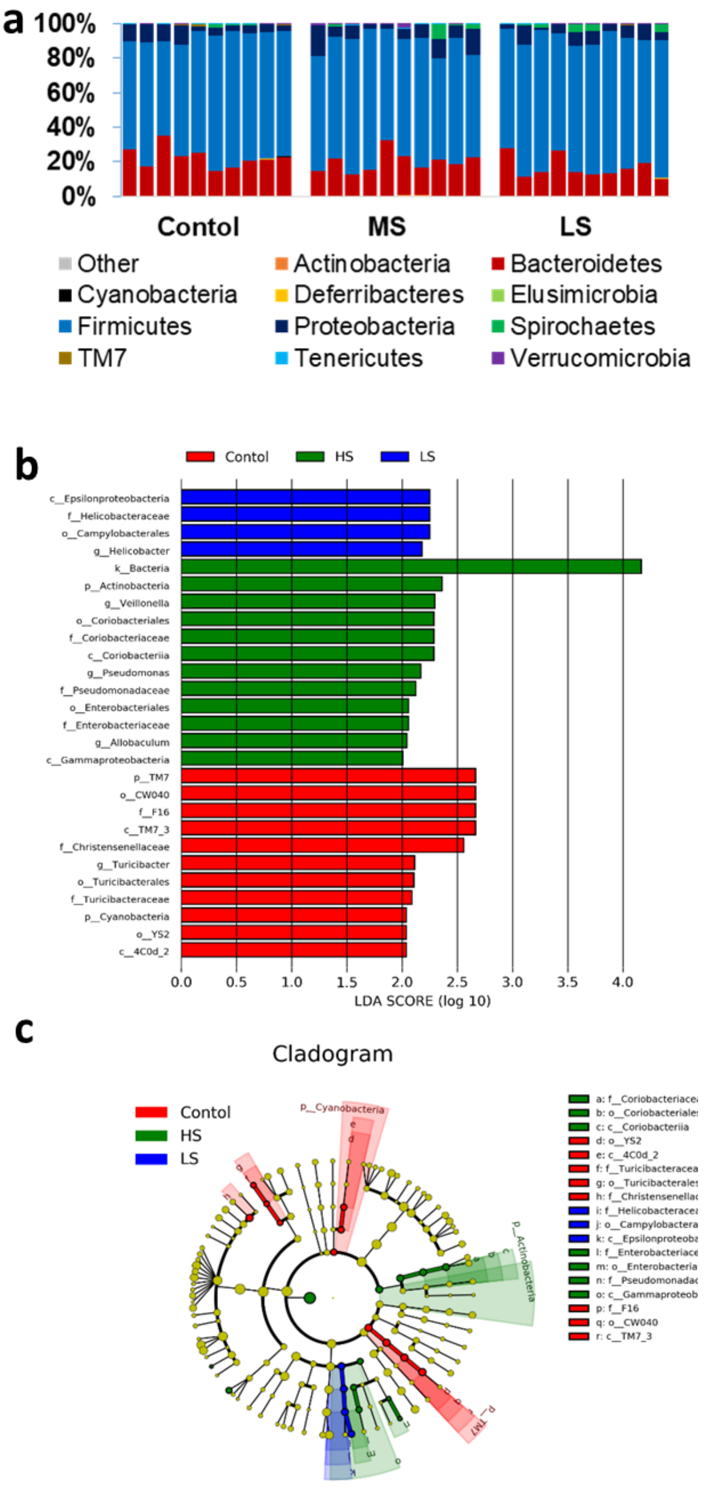

Supplementary Figure 1. Salt intake for 4 weeks structured the gut microbiota profile. The composition and relative abundance at the phylum levels in the faecal microbiota community of the control, medium salt (MS, 4% NaCl), and high salt (HS, 8% NaCl) gerbils (a). Differential bacterial taxonomy selected by LEfSe analysis in the faecal microbiota community (b). Cladogram representing taxa enriched in the faecal microbiota community of the 3 groups detected by the LEfSe tool with LDA >2(c).

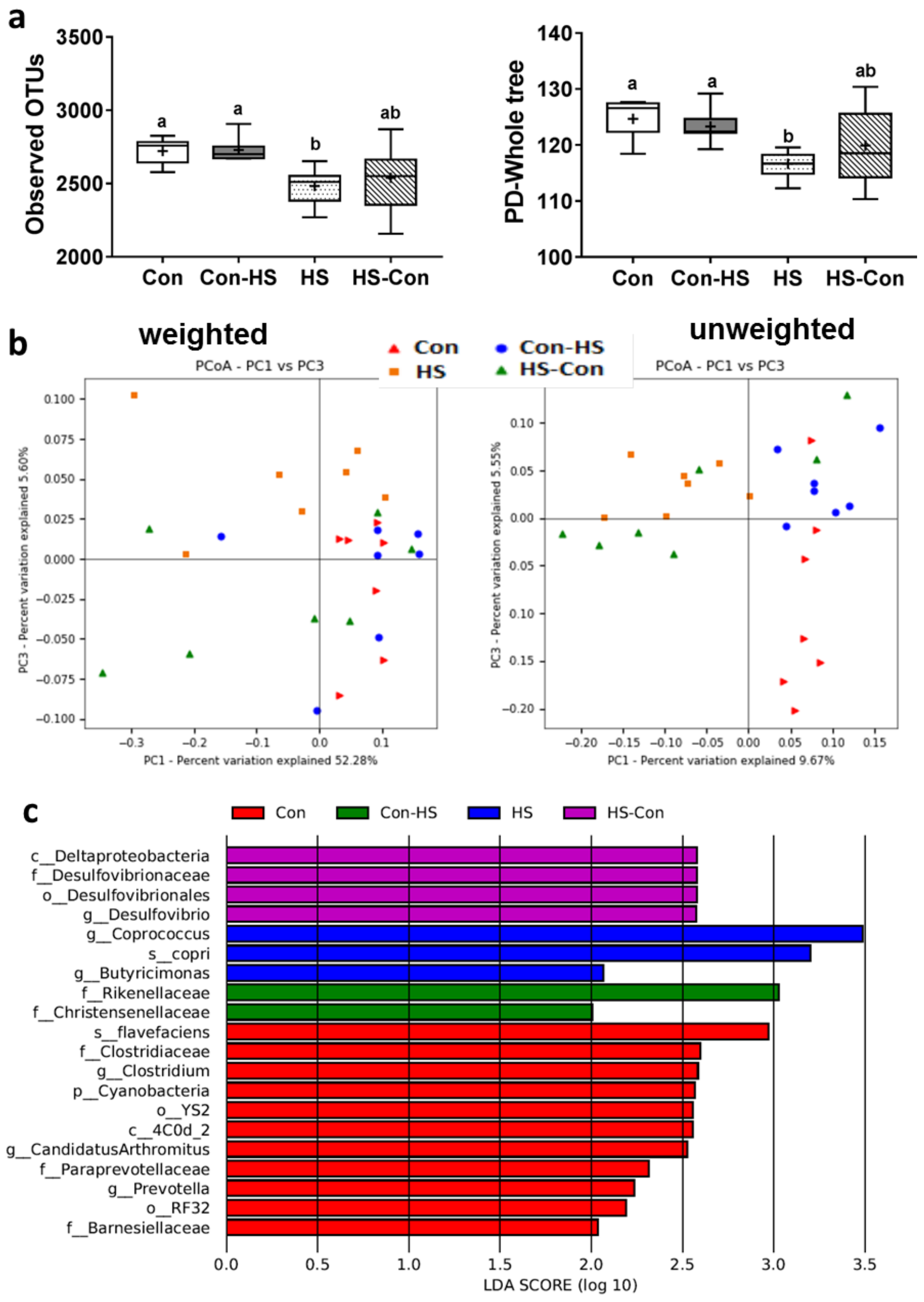

Supplementary Figure 2. The gut microbiota profile at the end of the 20-week period. Observed operation taxonomic units (OTUs, $P < 0.001$), and phylogenetic diversity (PD) whole tree ($P < 0.001$) (a); principal coordinate analyses (PCoA) plots based on weighted and unweighted UniFrac distances in the faecal microbiota of different groups (b); differential bacterial taxa selected by LEfSe analysis with LDA >2 in the faecal bacterial community (c). Data are presented as means ± s.e.m, and bars which do not share the same letter are significantly different from each other ($P < 0.05$). CMT, caecal microbiota transplant; Con, gerbils drank tap water *ad libitum* and received sterile saline as sham-CMT via oral gavage; Con-HS, Con gerbils received microbiota from HS gerbils via oral gavage; HS, gerbils drank water *ad libitum* with 8% NaCl and received sterile saline as sham-CMT via oral gavage; HS-Con, gerbils drank HS water *ad libitum* and received microbiota from the Con gerbils via oral gavage.

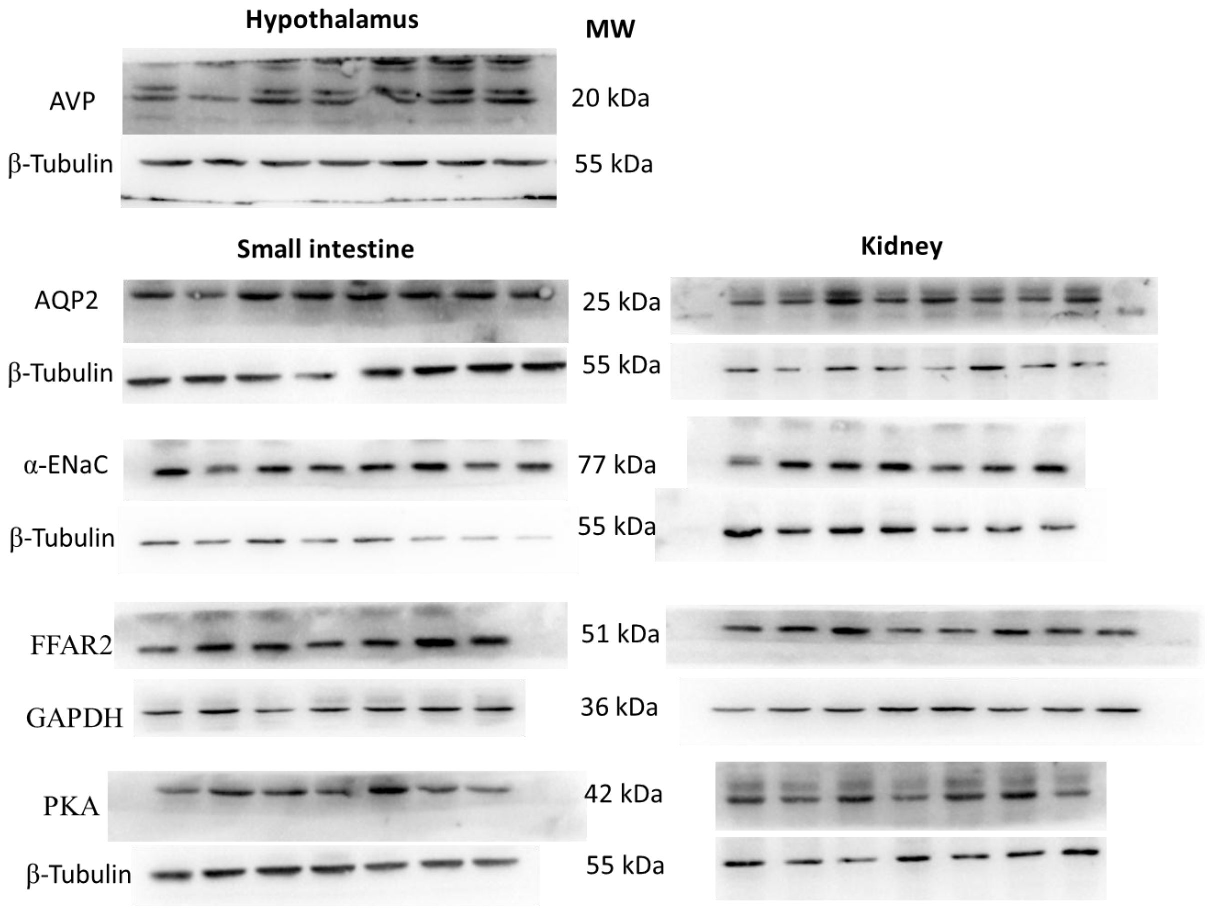

Supplementary Figure 3. The unprocessed scans of the most important blots. Arginine vasopressin (AVP) in the hypothalamus; aquaporin 2 (AQP2) in small intestine and kidney; epithelial sodium channel (α-ENaC) in small intestine and kidney; free fatty acid receptor 2 (FFAR2) in small intestine and kidney; and cAMP-activated protein kinase (PKA) in small intestine and kidney. MW, molecular weight. The protein marker covering the expected molecular weight range was loaded to the wells on both sides for later accurately cutting of the gels and estimation of the detected proteins. All blots derived from the same experiment and were processed in parallel.

Supplementary QIIME and R Codes

cho "Denoising and chimera detection using usearch61"

identify_chimeric_seqs.py -i 2-SplitLibrary/seqs.fna --threads=2 -m usearch61 -o 3-OTUs/usearch_checked_chimeras -r /share/disk3/wangjf/software/Tools/python2.7/lib/python2.7/site-packages/qiime_default_reference-0.1.1-py2.7.egg/qiime_default_reference/gg_13_8_otus/rep_set/97_otus.fasta

filter_fasta.py -f 2-SplitLibrary/seqs.fna -o 3-OTUs/seqs_chimeras_filtered.fna -s 3-OTUs/usearch_checked_chimeras/chimeras.txt -n

echo "Pick OTUs"

pick_open_reference_otus.py -i 3-OTUs/seqs_chimeras_filtered.fna -o 3-OTUs/uclust_picked_otus

filter_taxa_from_otu_table.py -i 3-OTUs/uclust_picked_otus/otu_table_mc2_w_tax.biom -p k__Bacteria -o 3-OTUs/uclust_picked_otus/otu_table_mc2_w_tax_filter.biom

echo "Summarize taxa by levels"

echo "summarize_taxa:level 1,2,3,4,5,6,7" > summarize_params.txt

echo "plot_taxa_summary:chart_type area,bar,pie" >> summarize_params.txt

summarize_taxa_through_plots.py -i 3-OTUs/uclust_picked_otus/otu_table_mc2_w_tax.biom -o 3-OTUs/taxa_summary -m 2_Mapping.txt -p summarize_params.txt

echo "OTU Network"

make_otu_network.py -m 2_Mapping.txt -i 3-OTUs/uclust_picked_otus/otu_table_mc2_w_tax_filter.biom -o 3-OTUs/OTU_Network

echo "Summarize taxa"

summarize_taxa_through_plots.py -i 3-OTUs/uclust_picked_otus/otu_table_mc2_w_tax_filter.biom -o 4-Taxa_summary -m 2_Mapping.txt -p summarize_params.txt

echo "Alpha rarefaction"

echo "alpha_diversity:metrics shannon,PD_whole_tree,chao1,observed_species,observed_otus" > alpha_params.txt

alpha_rarefaction.py -i 3-OTUs/uclust_picked_otus/otu_table_mc2_w_tax_filter.biom -m 2_Mapping.txt -o 5-AlphaRarefaction -t 3-OTUs/uclust_picked_otus/rep_set.tre -p alpha_params.txt

echo "Beta diversity and plots"

beta_diversity_through_plots.py -i 3-OTUs/uclust_picked_otus/otu_table_mc2_w_tax_filter.biom -m 2_Mapping.txt -t 3-OTUs/uclust_picked_otus/rep_set.tre -e 12000 -o 6-BetaDiversity

echo "Comparing Categories using ANOSIM"

compare_categories.py --method anosim -i 6-BetaDiversity/unweighted_unifrac_dm.txt -m 2_Mapping.txt -c Type -o 6-BetaDiversity/unweighted_unifrac_Type_anosim -n 999

compare_categories.py --method anosim -i 6-BetaDiversity/weighted_unifrac_dm.txt -m 2_Mapping.txt -c Type -o 6-BetaDiversity/weighted_unifrac_Type_anosim -n 999

echo "Jackknifed beta diversity"

jackknifed_beta_diversity.py -i 3-OTUs/uclust_picked_otus/otu_table_mc2_w_tax_filter.biom -t 3-OTUs/uclust_picked_otus/rep_set.tre -m 2_Mapping.txt -e 12000 -o 7-Jacknifed

echo "Make Bootstrapped Tree"

make_bootstrapped_tree.py -m 7-Jacknifed/unweighted_unifrac/upgma_cmp/master_tree.tre -s 7-Jacknifed/unweighted_unifrac/upgma_cmp/jackknife_support.txt -o 7-Jacknifed/unweighted_unifrac/upgma_cmp/jackknife_named_nodes.pdf

echo "Make Bi-Plots"

make_emperor.py -m 2_Mapping.txt -i 6-BetaDiversity/unweighted_unifrac_pc.txt -t 4-Taxa_summary/otu_table_mc2_w_tax_filter_L3.txt --n_taxa_to_keep 5 -o 8-Biplots

echo "Cluster Analysis"

mkdir 9-ClusterAnalysis

metaphlan_hclust_heatmap.py --in 9-ClusterAnalysis/Taxa.xls --out 9-ClusterAnalysis/phyla_abundance_heatmap.png -c bbcry --top 10 --minv 0.0001 -s log --tax_lev p

metaphlan_hclust_heatmap.py --in 9-ClusterAnalysis/Taxa.xls --out 9-ClusterAnalysis/class_abundance_heatmap.png -c bbcry --top 20 --minv 0.0001 -s log --tax_lev c

metaphlan_hclust_heatmap.py --in 9-ClusterAnalysis/Taxa.xls --out 9-ClusterAnalysis/orders_abundance_heatmap.png -c bbcry --top 30 --minv 0.0001 -s log --tax_lev o

metaphlan_hclust_heatmap.py --in 9-ClusterAnalysis/Taxa.xls --out 9-ClusterAnalysis/families_abundance_heatmap.png -c bbcry --top 40 --minv 0.0001 -s log --tax_lev f

metaphlan_hclust_heatmap.py --in 9-ClusterAnalysis/Taxa.xls --out 9-ClusterAnalysis/genera_abundance_heatmap.png -c bbcry --top 50 --minv 0.0001 -s log --tax_lev g

metaphlan_hclust_heatmap.py --in 9-ClusterAnalysis/Taxa.xls --out 9-ClusterAnalysis/species_abundance_heatmap.png -c bbcry --top 50 --minv 0.0001 -s log --tax_lev s

echo "High-Dimensional biomarker discovery and explanation using LEfSe"

mkdir 10-Biomarker

format_input.py Taxa.xls Type.in -c 1 -s 2 -u 3 -o 1000000

run_lefse.py Type.in Type.out -l 2 -y 0

plot_res.py Type.out Type.lefse_biomarker.png --feature_font_size 10 --width 10 --dpi 300 --format png --left_space 0.3

plot_cladogram.py Type.out Type.lefse_biomarkers_cladogram.png --class_legend_font_size 6.5 --dpi 300 --format png

plot_features.py Type.in Type.out Type.biomarkers.zip --archive zip --format png

echo "Function Analysis"

mkdir 11-Function

filter_otus_from_otu_table.py -i 3-OTUs/uclust_picked_otus/otu_table_mc2_w_tax_filter.biom -o 11-Function/closed_otu_table.biom --negate_ids_to_exclude -e /share/disk3/wangjf/software/Tools/python2.7/lib/python2.7/site-packages/qiime_default_reference-0.1.3-py2.7.egg/qiime_default_reference/g

g_13_8_otus/rep_set/97_otus.fasta

normalize_by_copy_number.py -i 11-Function/closed_otu_table.biom -o 11-Function/normalized_otus.biom

predict_metagenomes.py -i 11-Function/normalized_otus.biom -o 11-Function/metagenome_predictions_ko.biom

mkdir 11-Function/KEGG

categorize_by_function.py -i 11-Function/metagenome_predictions_ko.biom -c KEGG_Pathways -l 3 -o 11-Function/KEGG/predicted_metagenomes.L3.biom

categorize_by_function.py -i 11-Function/metagenome_predictions_ko.biom -c KEGG_Pathways -l 3 -o 11-Function/KEGG/predicted_metagenomes.L3.txt -f

categorize_by_function.py -i 11-Function/metagenome_predictions_ko.biom -c KEGG_Pathways -l 2 -o 11-Function/KEGG/predicted_metagenomes.L2.biom

categorize_by_function.py -i 11-Function/metagenome_predictions_ko.biom -c KEGG_Pathways -l 2 -o 11-Function/KEGG/predicted_metagenomes.L2.txt -f

categorize_by_function.py -i 11-Function/metagenome_predictions_ko.biom -c KEGG_Pathways -l 1 -o 11-Function/KEGG/predicted_metagenomes.L1.biom

categorize_by_function.py -i 11-Function/metagenome_predictions_ko.biom -c KEGG_Pathways -l 1 -o 11-Function/KEGG/predicted_metagenomes.L1.txt -f

predict_metagenomes.py -i 11-Function/normalized_otus.biom -o 11-Function/metagenome_predictions_cog.biom -t cog

mkdir 11-Function/COG

categorize_by_function.py -i 11-Function/metagenome_predictions_cog.biom -c COG_Category -l 2 -o 11-Function/COG/predicted_metagenomes.L2.biom

categorize_by_function.py -i 11-Function/metagenome_predictions_cog.biom -c COG_Category -l 2 -o 11-Function/COG/predicted_metagenomes.L2.txt -f

categorize_by_function.py -i 11-Function/metagenome_predictions_cog.biom -c COG_Category -l 1 -o 11-Function/COG/predicted_metagenomes.L1.biom

categorize_by_function.py -i 11-Function/metagenome_predictions_cog.biom -c COG_Category -l 1 -o 11-Function/COG/predicted_metagenomes.L1.txt –f
