# Supplementary material for: The microbiota-gut-kidney axis mediates host osmoregulation in a small desert mammal
Source: NPJ Biofilms Microbiomes. 2022 Apr 4;8:16. doi: 10.1038/s41522-022-00280-5 (PMC8980004; doi:10.1038/s41522-022-00280-5)
Supplement: Supplementary file 2 — Reporting summary [file 41522_2022_280_MOESM2_ESM.pdf]

nature portfolio

Corresponding author(s): Dehua Wang; Xueying Zhang

Last updated by author(s): Jan 22, 2022

# Reporting Summary

Nature Portfolio wishes to improve the reproducibility of the work that we publish. This form provides structure for consistency and transparency in reporting. For further information on Nature Portfolio policies, see our Editorial Policies and the Editorial Policy Checklist.

## Statistics

For all statistical analyses, confirm that the following items are present in the figure legend, table legend, main text, or Methods section.

| n/a | Confirmed | |
|---|---|---|
| ☐ | ☒ | The exact sample size ($n$) for each experimental group/condition, given as a discrete number and unit of measurement |
| ☐ | ☒ | A statement on whether measurements were taken from distinct samples or whether the same sample was measured repeatedly |
| ☐ | ☒ | The statistical test(s) used AND whether they are one- or two-sided<br>*Only common tests should be described solely by name; describe more complex techniques in the Methods section.* |
| ☐ | ☒ | A description of all covariates tested |
| ☐ | ☒ | A description of any assumptions or corrections, such as tests of normality and adjustment for multiple comparisons |
| ☐ | ☒ | A full description of the statistical parameters including central tendency (e.g. means) or other basic estimates (e.g. regression coefficient) AND variation (e.g. standard deviation) or associated estimates of uncertainty (e.g. confidence intervals) |
| ☐ | ☒ | For null hypothesis testing, the test statistic (e.g. $F$, $t$, $r$) with confidence intervals, effect sizes, degrees of freedom and $P$ value noted<br>*Give P values as exact values whenever suitable.* |
| ☒ | ☐ | For Bayesian analysis, information on the choice of priors and Markov chain Monte Carlo settings |
| ☐ | ☒ | For hierarchical and complex designs, identification of the appropriate level for tests and full reporting of outcomes |
| ☒ | ☐ | Estimates of effect sizes (e.g. Cohen's $d$, Pearson's $r$), indicating how they were calculated |

*Our web collection on statistics for biologists contains articles on many of the points above.*

## Software and code

Policy information about availability of computer code

| | |
|---|---|
| Data collection | n/a |
| Data analysis | QIIME and R codes that were used in the 16S rRNA gene amplicon sequencing and analysis are available in the supplementary file. |

For manuscripts utilizing custom algorithms or software that are central to the research but not yet described in published literature, software must be made available to editors and reviewers. We strongly encourage code deposition in a community repository (e.g. GitHub). See the Nature Portfolio guidelines for submitting code & software for further information.

## Data

Policy information about availability of data

All manuscripts must include a data availability statement. This statement should provide the following information, where applicable:

- Accession codes, unique identifiers, or web links for publicly available datasets
- A description of any restrictions on data availability
- For clinical datasets or third party data, please ensure that the statement adheres to our policy

The raw data of 16S rRNA gene amplicon sequence are available in the NCBI Sequence Read Archive under accession PRJNA759876 and PRJNA796757.

# Field-specific reporting

Please select the one below that is the best fit for your research. If you are not sure, read the appropriate sections before making your selection.

[x] Life sciences    [ ] Behavioural & social sciences    [ ] Ecological, evolutionary & environmental sciences

For a reference copy of the document with all sections, see nature.com/documents/nr-reporting-summary-flat.pdf

# Life sciences study design

All studies must disclose on these points even when the disclosure is negative.

| | |
|---|---|
| Sample size | The sample size is at least 8 in each group which is usually sufficient in physiological studies. |
| Data exclusions | No data were excluded from the analysis. |
| Replication | The measures were taken to verify the reproducibility of the experimental findings. |
| Randomization | The animals were allocated into experimental groups randomly. |
| Blinding | The investigators were blinded to group collection during data collection and analysis. |

# Reporting for specific materials, systems and methods

We require information from authors about some types of materials, experimental systems and methods used in many studies. Here, indicate whether each material, system or method listed is relevant to your study. If you are not sure if a list item applies to your research, read the appropriate section before selecting a response.

## Materials & experimental systems

| n/a | Involved in the study | |
|---|---|---|
| [ ] | [x] | Antibodies |
| [x] | [ ] | Eukaryotic cell lines |
| [x] | [ ] | Palaeontology and archaeology |
| [ ] | [x] | Animals and other organisms |
| [x] | [ ] | Human research participants |
| [x] | [ ] | Clinical data |
| [x] | [ ] | Dual use research of concern |

## Methods

| n/a | Involved in the study | |
|---|---|---|
| [x] | [ ] | ChIP-seq |
| [x] | [ ] | Flow cytometry |
| [x] | [ ] | MRI-based neuroimaging |

## Antibodies

| | |
|---|---|
| Antibodies used | anti-AVP antibody (AB1565; Merck Millipore, Darmstadt, Germany), anti-AQP2 antibody (sc-9882; Santa Cruz Biotechnology, CA, USA), anti-[illegible]-ENaC antibody (PA1-920A; Thermo Fisher, Waltham, MA, USA), anti-FFAR2/GPR43 antibody (ABC299; Merck Millipore, Darmstadt, Germany), anti-cAMP protein kinase catalytic subunit antibody (ab26322; Abcam, Cambridge, UK), anti-β-Tubulin antibody (30301ES60; Yeasen, Shanghai, China) and anti-GAPDH antibody (30201ES60; Yeasen) |
| Validation | The antibodies were validated previously and the references were cited. |

## Animals and other organisms

Policy information about studies involving animals; ARRIVE guidelines recommended for reporting animal research

| | |
|---|---|
| Laboratory animals | Mongolian gerbils (Meriones unguiculatus),Females,aged 6 to 12 months |
| Wild animals | The breeding colonies of Mongolian gerbils in the laboratory at the Institute of Zoology, the Chinese Academy of Sciences |
| Field-collected samples | No field-collected samples. |
| Ethics oversight | The protocol and procedures in this study were approved by the Animal Care and Use Committee of the Institute of Zoology. |

Note that full information on the approval of the study protocol must also be provided in the manuscript.
